# Supplementary material for: Giant single molecule chemistry events observed from a tetrachloroaurate(III) embedded Mycobacterium smegmatis porin A nanopore
Source: Nat Commun. 2019 Dec 11;10:5668. doi: 10.1038/s41467-019-13677-2 (PMC6906327; doi:10.1038/s41467-019-13677-2)
Supplement: Supplementary file 1 — Supplementary Information [file 41467_2019_13677_MOESM1_ESM.pdf]

Supplementary Information

Giant single molecule chemistry events observed from a tetrachloroaurate(III) embedded *Mycobacterium smegmatis* porin A nanopore

Cao *et al.*

List of Content

Supplementary Figure 1-19

Supplementary Table 1-10

Supplementary References

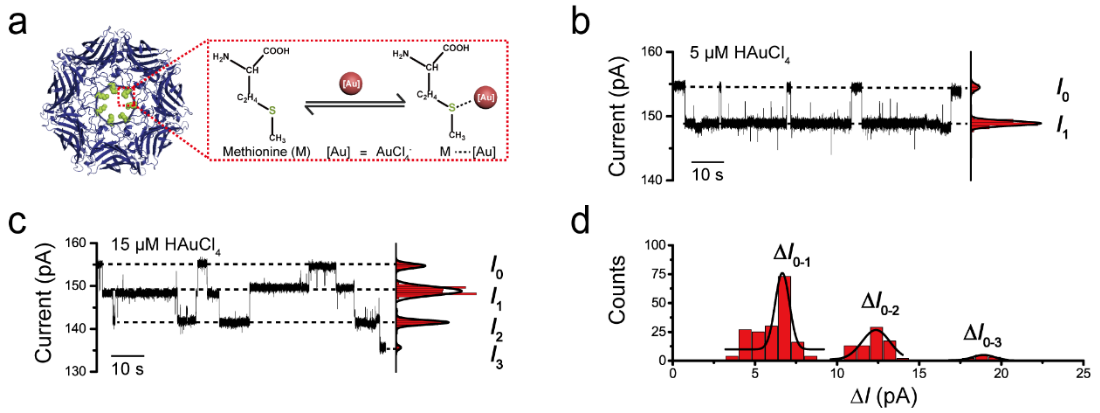

Supplementary Figure 1. $[AuCl_4]^-$ binding within a WT α-HL nanopore. (a) Heptameric WT α-HL and its sensing mechanism for $[AuCl_4]^-$. Each heptameric WT α-HL nanopore has seven identical methionine residues at site 113 (yellow in the structural diagram). A reversible Au(III)-thioether coordination interaction takes place between a single $[AuCl_4]^-$ and any one of the seven methionine (M113) residues. **(b)** A representative trace of single $[AuCl_4]^-$ binding in WT α-HL with the corresponding all-points histogram. The electrophysiology recording was carried out by continuously applying a +100 mV voltage. $HAuCl_4$ was placed in *cis* with a 5 μM final concentration **(Methods)**. Continuous pore blockages by single $[AuCl_4]^-$ were clearly resolved with a consistent blockage amplitude (ΔI ≈6 pA). Though barely noticeable, some secondary state transitions were happening in the $[AuCl_4]^-$ bound state, indicating that non-specific interactions between $[AuCl_4]^-$ and other amino acid residues may exist. **(c)** A representative trace of multiple $[AuCl_4]^-$ binding in WT α-HL with the corresponding all-points histogram. The electrophysiology recording was carried out identically as described in **b (Methods)**. However, $HAuCl_4$ was placed in *cis* with a 15 μM final concentration. A high concentration of $[AuCl_4]^-$ in *cis* leads to sequential binding from multiple $[AuCl_4]^-$ within the same WT α-HL, which has resulted in multiple blockage states. Here, I_n stands for the state of n $[AuCl_4]^-$ currently bound in the pore. Direct state transition were strictly restricted between I_n and $I_{n\pm1}$. A direct transition between I_n and $I_{n\pm2}$ has never been observed. **(d)** The event histogram for $[AuCl_4]^-$ binding events. The statistical data was from a 10 min recording with a WT α-HL when 15 μM $HAuCl_4$ in *cis* was placed. Gaussian fittings for the histogram were performed. From the fitting results, the transition amplitude were derived as ΔI_{0-1}: 6 ± 1 pA, N=179; ΔI_{0-2}:12 ± 1 pA, N=75; ΔI_{0-3}:19 ± 1 pA, N=12. Here, ΔI_{0-n} stands for the current difference between I_0 and I_n respectively.

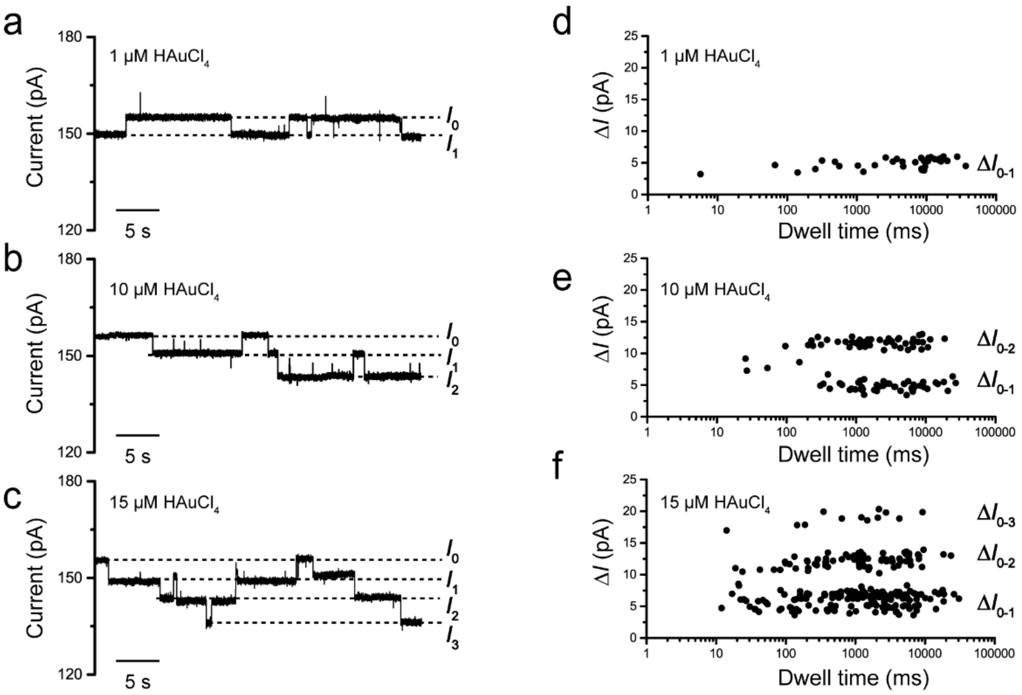

Supplementary Figure 2. Sequential $[AuCl_4]^-$ binding within a WT α-HL nanopore. (a-c) Representative traces of $[AuCl_4]^-$ bindings with different $HAuCl_4$ concentrations in *cis*. All electrophysiology recordings were carried out with a WT α-HL nanopore and a +100 mV voltage was applied **(Methods)**. 5-15 μM $HAuCl_4$ were placed in *cis* respectively. **(d-f)** The corresponding scatter plot of ΔI vs. the dwell time. The statistical data for all scatter plots were from 10 min continuous electrophysiology recordings for each condition. A wide dispersion of ΔI is clearly observed in **d**, **e** and **f**.

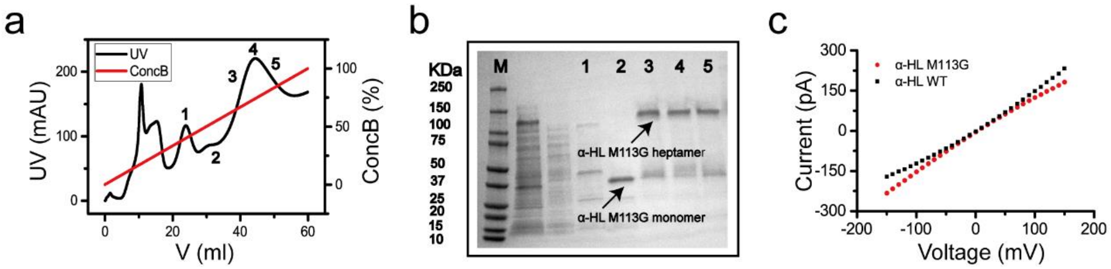

Supplementary Figure 3. Purification and characterization of hetpameric α-HL M113G. Monomeric α-HL M113G was expressed with *E Coli.* BL21 (DE3)[1]. Heptameric α-HL M113G spontaneously forms after cell lysis. The monomeric and heptameric α-HL M113G were isolated based on their different binding affinities with the nickel column. **(a)** The UV absorbance spectrum during column elution. The supernatant of the cell lysate was loaded onto a nickel affinity column and eluted with a gradient of imidazole (0 to 300 mM). The identities of the eluted samples were determined according to the corresponding gel electrophoresis. **(b)** Gel electrophoresis results for the eluted fractions. The gel electrophoresis was carried out with a 7.5% SDS-polyacrylamide gel. Lanes: M, precision plus protein standards (Bio-Rad); 1-5, the corresponding elution fractions in **(a)**. Based on the band positions in reference to previously published results of α-HL WT[1], fraction 2 was confirmed to contain monomeric α-HL M113G and fraction 3, 4, 5 were confirmed to contain the desired, heptameric α-HL M113G. Fraction 3-5 could be directly used for nanopore measurements or stored at -80 ℃ for long term storage. **(c)** IV curves for heptameric α-HL WT and α-HL M113G. Both IV curves were collected with the electrolyte buffer composed of 1.5 M KCl, 10 mM Tris-HCl, pH=7.0. Slight variations of IV curves between α-HL WT and M113G were systematically observed whereas the general conductance of the M113G mutant remains.

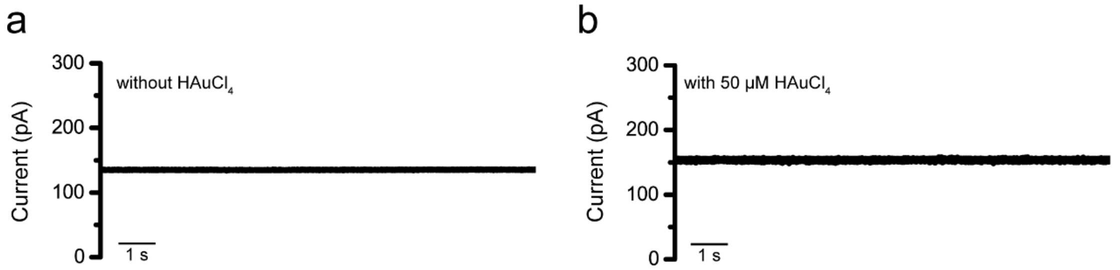

Supplementary Figure 4. The background current of α-HL M113G. The background current was recorded with α-HL M113G without or with $HAuCl_4$ in *cis* when a +100 mV voltage was applied **(Methods)**. **(a)** The background current recorded using α-HL M113G without $HAuCl_4$. With a +100 mV applied voltage, α-HL M113G stays open with no spontaneous gating, which indicates that an unaltered heptameric pore assembly forms after the mutation. **(b)** The background current recorded using α-HL M113G with 50 μM $HAuCl_4$ in *cis*. The measurement was performed with a +100 mV voltage and no $[AuCl_4]^-$ binding events were observed as the side group of the introduced glycine (M113G) doesn't establish any detectable interaction with tetrachloroaurate(III). This phenomenon indicates that methionine 113, which naturally exist in the WT α-HL, plays a critical role in tetrachloroaurate(III) binding, as observed in **Supplementary Figure 1, 2**.

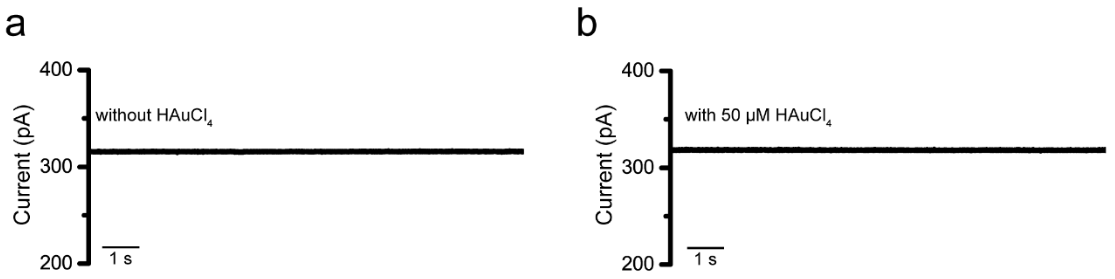

Supplementary Figure 5. The background current of M2 MspA. The background current was recorded with M2 MspA without or with $HAuCl_4$ in *cis* when a +100 mV voltage was continuously applied **(Methods)**. **(a)** The background current recorded without $HAuCl_4$ addition. No spontaneous gating of the pore was observed. **(b)** The background current with 50 µM $HAuCl_4$ in *cis*. No spontaneous gating or $[AuCl_4]^-$ binding event were observed. This phenomenon was expected as the M2 MspA mutant possesses no sulfur-containing amino acids (methionine or cysteine) in the vicinity of its pore restriction. Thus, M2 MspA, which is free from $[AuCl_4]^-$ binding ability, serves as an ideal pore engineering template for methionine introduction.

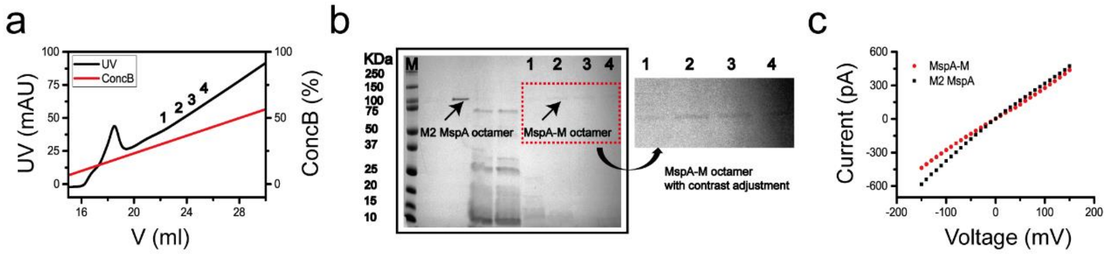

Supplementary Figure 6. Purification and characterization of octameric MspA-M. Monomeric MspA-M was expressed with *E Coli.* BL21 (DE3). Octameric MspA-M spontaneously forms immediately after cell lysis and was subsequently purified by nickel affinity chromatography **(Methods)**[1]. **(a)** The UV absorbance spectrum during column elution. The supernatant of the cell lysate was loaded onto a nickel affinity column and eluted with a gradient of imidazole (0 to 300 mM). Identities of the eluted samples were determined in the corresponding gel electrophoresis. The peak in the spectrum, which appears between the elution volume of 16 and 20 mL, was from proteins in the cell lysate non-specifically bound with the column. Though barely noticeable, the fractions which were marked with 1-4, were expected to contain the octameric MspA-M during elution, based on our previous experience with M2 MspA purification[1]. **(b)** Gel electrophoresis results for different elution fractions. The gel electrophoresis was performed with a 7.5% SDS-polyacrylamide gel. Lanes: M, precision plus protein standards (Bio-Rad); 1-4, the corresponding elution fractions in **(a)**. Although barely visible, fractions 1-3 were confirmed to contain octameric MspA-M by taking M2 MspA as a reference. The purified octameric MspA-M could be immediately used in downstream nanopore measurements or stored at -80 ℃ for long term storage. The image inset was contrast adjusted so that the band for the octameric MspA-M could be demonstrated. **(c)** IV curves of M2 MspA and MspA-M nanopore. Both IV curves were collected with the electrolyte buffer composed of 1.5 M KCl, 10 mM Tris-HCl, pH=7.0. Slight variations of IV curves between M2 MspA and MspA-M were systematically observed whereas the general conductance of MspA-M retains with no noticeable gating. These phenomena indicate that the monomeric MspA-M mutant has successfully assembled into its octameric form and a rigid pore geometry was maintained.

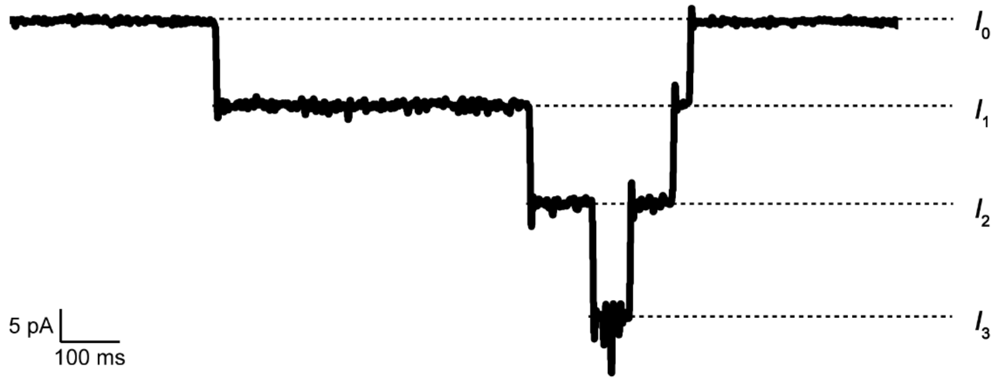

Supplementary Figure 7. Binding of multiple $[AuCl_4]^-$ ions in a MspA-M. Sequential and reversible binding of multiple $[AuCl_4]^-$ within the same MspA-M nanopore could be frequently observed when a high concentration of $HAuCl_4$ were placed in *cis*. The blockage levels, namely I_n (n=0-3), show an extremely high consistency in the amplitude. Here, n of I_n stands for n $[AuCl_4]^-$ simultaneously bound in the pore. Direct state transitions were restricted between I_n and $I_{n\pm1}$. Direct transitions between I_n and $I_{n\pm2}$ have never been observed. The electrophysiology recording was performed with MspA-M when a +100 mV applied voltage was continuously applied. $HAuCl_4$ was added in *cis* with a 10 µM final concentration. See **Supplementary Video 2** for a live trace playback.

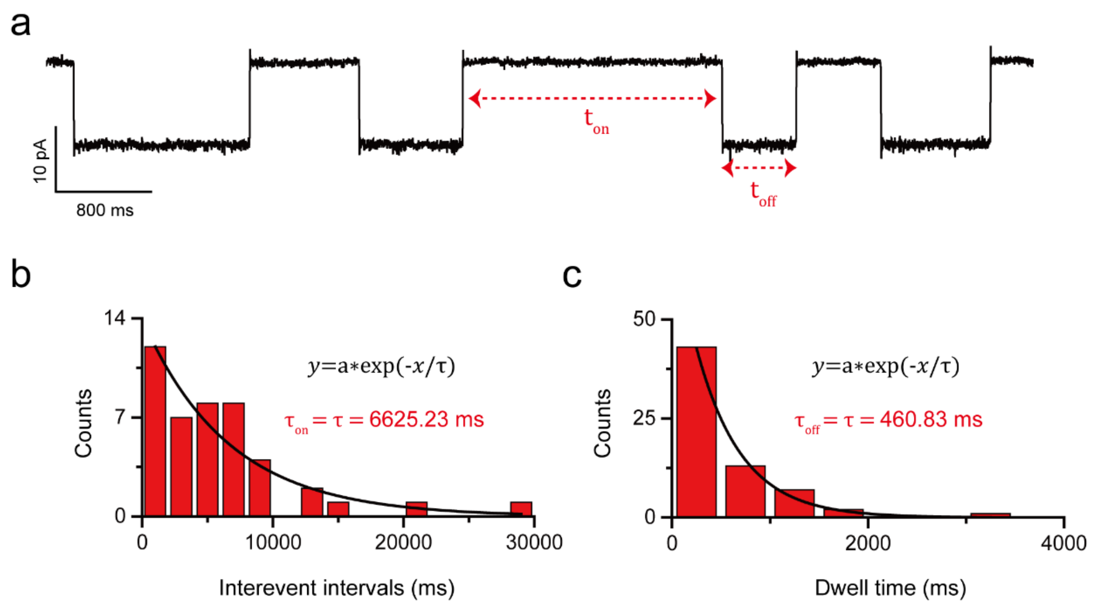

Supplementary Figure 8. Analysis of the dwell time and the inter-event intervals. (a) A representative electrophysiology trace of tetrachloroaurate(III) binding with a MspA-M nanopore. t_{on} represents the inter-event duration time. Whereas, t_{off} represents the event dwell time. **(b-c)** Histogram plots of the inter-event duration time (t_{on}) and the event dwell time (t_{off}) with corresponding single exponential fittings respectively. The single exponential fitting was performed according to the equation $\mathrm{y} = \mathrm{a} * \exp(-\mathrm{x}/\tau)$. The mean inter-event interval (τ_{on}) or the mean dwell time (τ_{off}) was derived from the fitting result respectively. All mean dwell time and inter-event intervals values in this paper were derived as described if not otherwise stated.

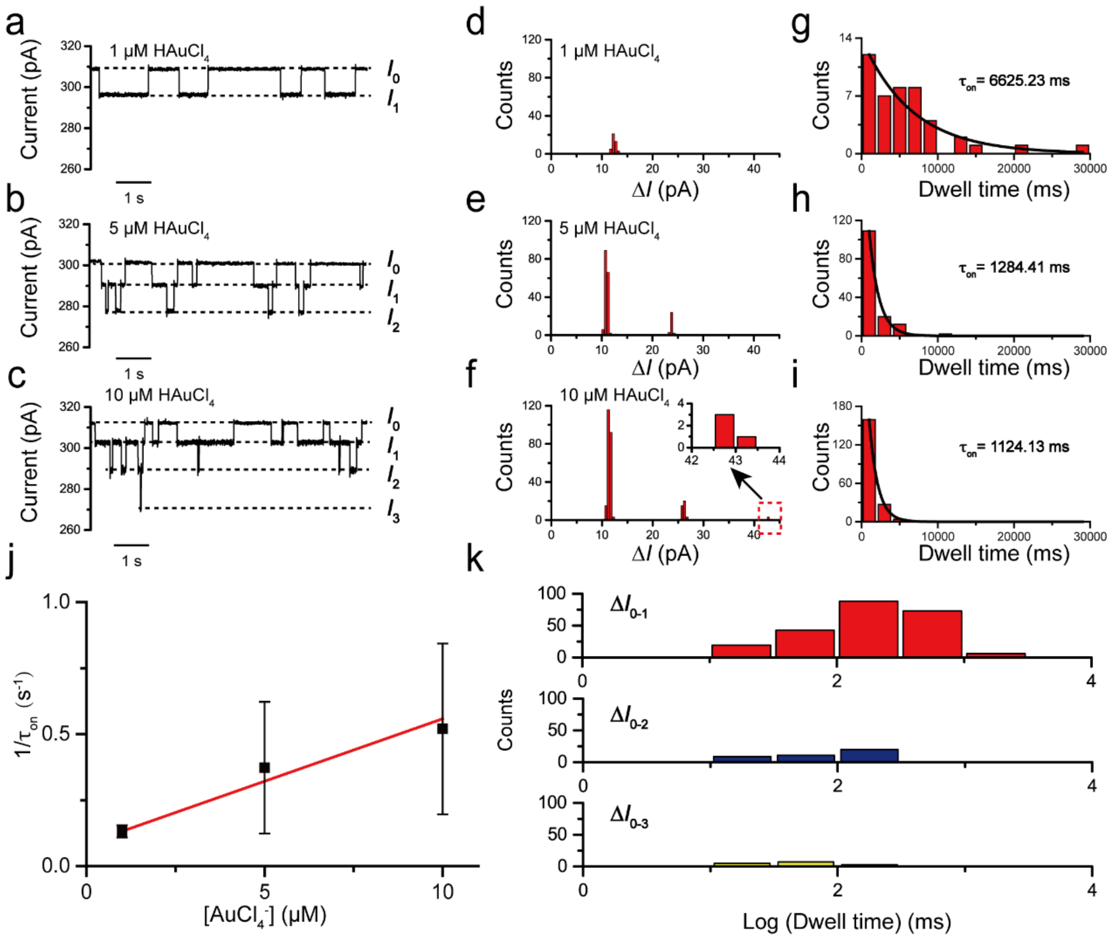

Supplementary Figure 9. $[AuCl_4]^-$ binding kinetics within an MspA-M nanopore. (a-c) Representative current recordings with different $HAuCl_4$ concentrations in *cis* (1 μM, 5 μM or 10 μM). The nanopore measurements were performed as described in Methods with a continuously applied voltage of +100 mV. **(d-f)** Corresponding blockage event histograms with different $HAuCl_4$ concentrations in *cis*. **(g-i)** The derivation of mean inter-event intervals (τ_{on}) with different $HAuCl_4$ concentrations in *cis*. All statistics **(d-i)** were performed from a continuous electrophysiology recording of 280 s for each condition. **(j)** Plot of the reciprocals of the mean inter-event intervals (τ_{on}) versus $HAuCl_4$ ions concentrations in *cis*. The $1/\tau_{on}$ values increase when the $HAuCl_4$ concentrations in *cis* was increased. This phenomena indicate that the observed blockage events do result from binding of tetrachloroaurate(III). **(k)** Log dwell-time histograms for ΔI_{0-1}, ΔI_{0-2}, and ΔI_{0-3} in the presence of 10 μM $HAuCl_4$ in *cis*. The much reduced mean dwell time for ΔI_{0-3} means that the probability for the pore to simultaneously accommodate 3 tetrachloroaurate(III) is small.

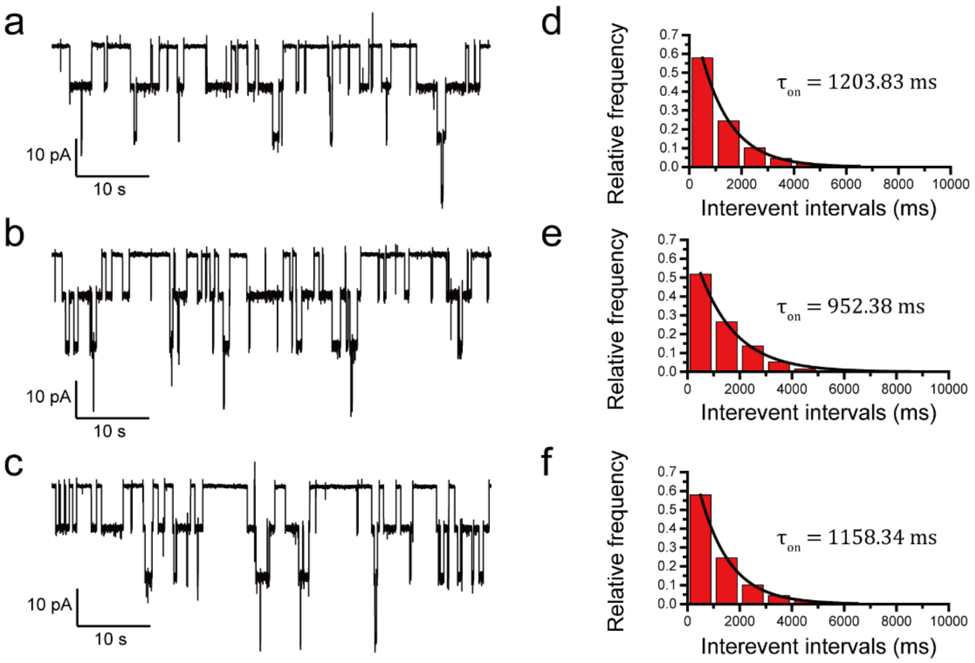

Supplementary Figure 10. Time extended observation of [AuCl4]- binding to MspA-M. Nanopore measurements were performed as described in **Methods**. $HAuCl_4$ was placed in *cis* with a 4 μM final concentration followed with 1 minute magnetic stirring. Electrophysiology recordings were acquired **(a)** immediately after, **(b)** one hour after and **(c)** two hours after $HAuCl_4$ placement when a +100 mV bias was continuously applied. **(d-f)** Histograms of mean inter-event intervals (τ_{on}). All statistics demonstrated in **(d-f)** were formed from results during a ten minutes continuous electrophysiology recording **(d)** immediately after, **(e)** one hour after and **(f)** two hours after $HAuCl_4$ placement. The derived mean inter-event intervals (τ_{on}) indicate that binding of $[AuCl_4]^-$ to MspA-M has reached the equilibrated state immediately after the $HAuCl_4$ placement.

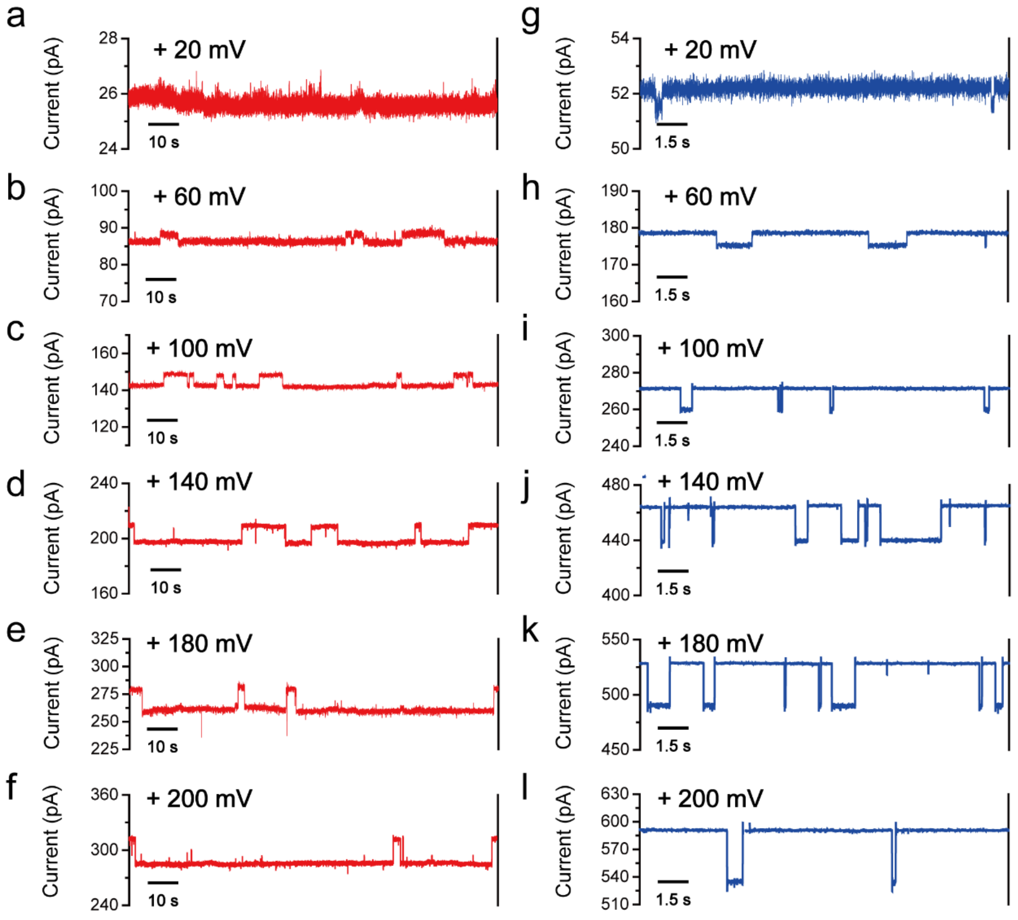

Supplementary Figure 11. $[AuCl_4]^-$ binding at different voltages. (a-f) Representative traces for $[AuCl_4]^-$ binding with WT α-HL when a +20 mV **(a)**, +60 mV **(b)**, +100 mV **(c),** +140 mV **(d),** +180 mV **(e)** and +200 mV **(f)** voltage was applied. $HAuCl_4$ in *cis* was kept with a 15 µM concentration. **(g-l)** Representative traces for $[AuCl_4]^-$ binding with MspA-M when a +20 mV **(g)**, +60 mV **(h)**, +100 mV **(i),** +140 mV **(j),** +180 mV **(k)** and +200 mV **(l)** voltage was applied. $HAuCl_4$ in *cis* was kept with a 1 µM concentration. All recorded traces **(a-l) (Methods)** were digitally filtered with a 200 Hz low-pass Bessel filter (eight-pole) in Clampfit so that the shallow binding events in **(a)** and **(g)** were presented. Baseline fluctuations were systematically observed in all electrophysiology recordings with WT α-HL whenever $[AuCl_4]^-$ were placed in the system. The fluctuation was more obviously observed when a high voltage (>100 mV) was applied **(c-f)**. These phenomena suggest that other amino acid residues such as K131 and K147[2], which distribute over the long, cylindrical restriction of WT α-HL, may have established non-desired interactions with $[AuCl_4]^-$. However, the conical geometry of the MspA nanopore has much reduced the interference from other non-desired interactions away from the restriction to be reported.

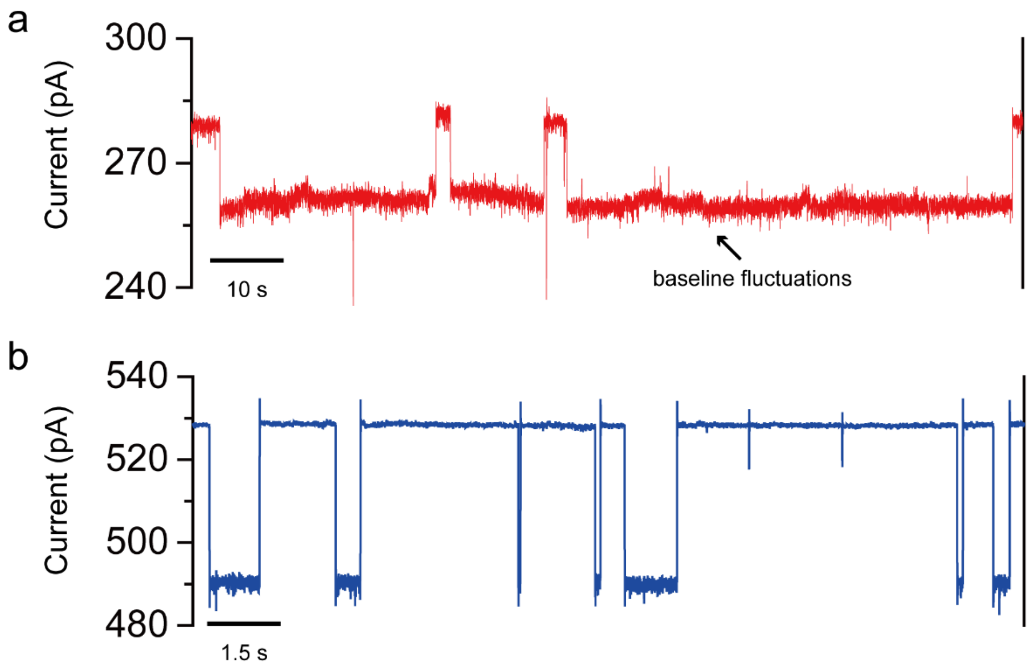

Supplementary Figure 12. $[AuCl_4]^-$ binding with WT α-HL and MspA-M at +180 mV. (a) A representative electrophysiology trace for $[AuCl_4]^-$ binding with WT α-HL when a +180 mV voltage was continuously applied. $HAuCl_4$ in *cis* was kept with a 15 μM final concentration. Baseline fluctuations were systematically observed in electrophysiology recordings whenever $HAuCl_4$ were placed in *cis* and a high voltage (> +100 mV) was applied. **(b)** A representative electrophysiology trace for $[AuCl_4]^-$ binding with MspA-M when a +180 mV voltage was applied. $HAuCl_4$ was placed in *cis* with a 1 μM final concentration. No noticeable baseline fluctuations were observed from the electrophysiology trace, even with a high applied voltage (+180 mV). The blockage signal of $[AuCl_4]^-$ binding appear flat and consistent. This phenomenon suggests that there is only one type of interaction that exist between $[AuCl_4]^-$ and MspA-M, around the vicinity of the pore restriction.

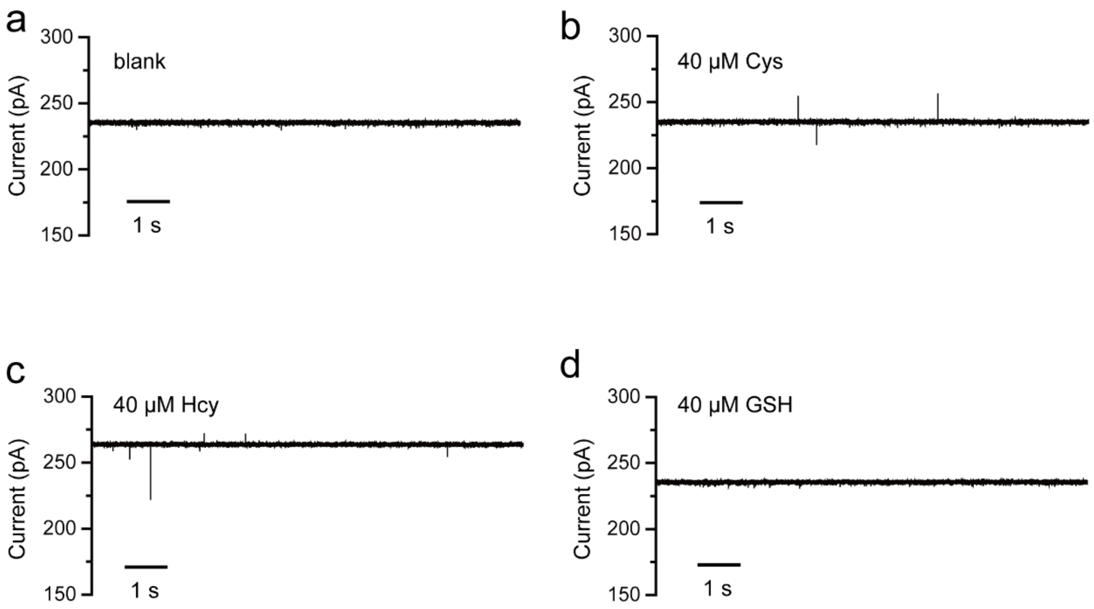

Supplementary Figure 13. MspA-M and biothiols. The background current was recorded with MspA-M when a +100 mV voltage was continuously applied **(Methods)**. No $HAuCl_4$ was added in *cis* for this set of measurements. **(a)** The background current recorded using MspA-M without the addition of any biothiols. MspA-M stays open with no spontaneous gating activities in this condition. **(b-d)** The background current recorded using MspA-M with 40 μM Cys **(b)**, 40 μM Hcy **(c)** or 40 μM GSH **(d)** in *trans*. No Cys, Hcy or GSH binding event were observed. Without the Au(III) embedment as an atomic bridge, single molecule sensing of biothiols cannot be directly performed with MspA-M.

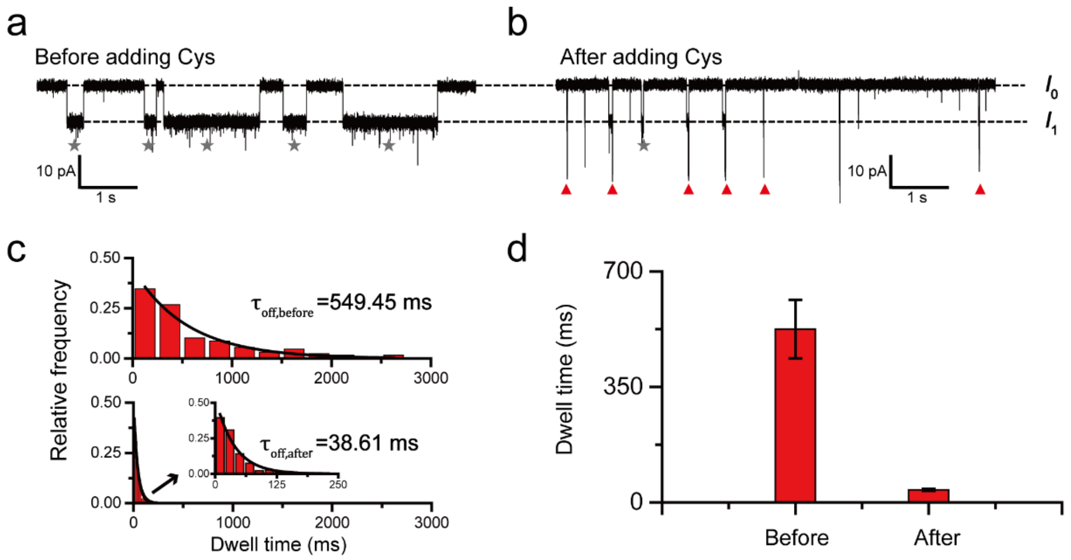

Supplementary Figure 14. The dwell time of state 1 after the addition of Cys. (a) A representative trace for single $[AuCl_4]^-$ binding with MspA-M when 4 µM $HAuCl_4$ were placed in *cis*. Grey stars mark the events of $[AuCl_4]^-$ binding. **(b)** A representative trace for $[AuCl_4]^-$ binding with MspA-M when 4 µM $HAuCl_4$ in *cis* and 40 µM Cys in *trans* were placed. Red triangles mark the signals of Cys. Whereas, the grey star marks the $[AuCl_4]^-$ binding event. Other unlabeled events are background signals **(Supplementary Figure 15)**. **(c)** The dwell time (t_{off}) histogram of single $[AuCl_4]^-$ binding before and after the addition of 40 µM Cys in *trans*. Due to the competitive binding of Au(III) with the thiol of the cysteine against the thioether of the methionine around the pore restriction, the dwell time of state 1 is greatly shortened. **(d)** The bar plot of the mean dwell time (τ_{off}) of single $[AuCl_4]^-$ binding before and after the addition of 40 µM Cys in *trans*. All means and standard deviations were from three independent experiments. (Before: 530 ± 90 ms; After: 38 ± 3 ms)

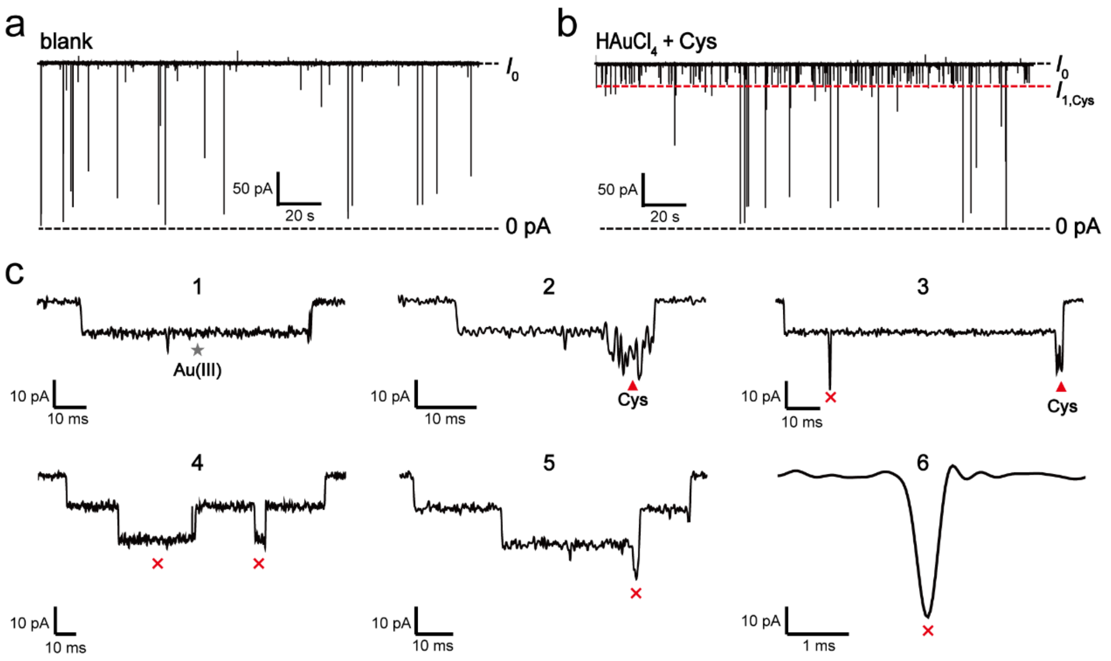

Supplementary Figure 15. Signal types of biothiols sensing. (a) The background current recorded using MspA-M without any analyte addition. Though no spontaneous gating was observed from MspA-M, during a long term of measurement with a +100 mV continuously applied voltage, transient pore blockages could still be observed. However, the blockage depth of these transient events is widely distributed. **(b)** A representative current trace recorded with MspA-M when a +100 mV voltage was continuously applied. 4 µM $HAuCl_4$ in *cis* and 40 µM Cys in *trans* were placed. The events of $[AuCl_4]^-$ or Cys binding show a narrow distribution of the blockage amplitude and could be easily discriminated from the background events. **(c)** A zoomed-in view of all possible event types during the measurement as described in **b**. Type 1 is a representative $[AuCl_4]^-$ binding event, as judged by its shape and blockage amplitude **(Fig. 1b)**. Type 1 event is excluded from the statistics of biothiols sensing as clearly no biothiol has bound with the pore when this event was acquired. Type 2 event is a representative biothiol event as discussed in **Fig. 3e** and is included in the statistics of biothiol sensing. Type 3 event is also a representative biothiol event. Transient spiky signals on top of the $[AuCl_4]^-$ binding state may be occasionally observed. Type 3 events were also included in the statistics. Type 4 and 5 events contains simultaneous binding of more than 1 $[AuCl_4]^-$ in the pore, as judged from the shape and the event blockage depth **(Supplementary Figure 7)**. To avoid complicating the statistics, these events were excluded from the statistics. Type 6 is the background event as observed in **a**. Type 6 events were excluded from the statistics.

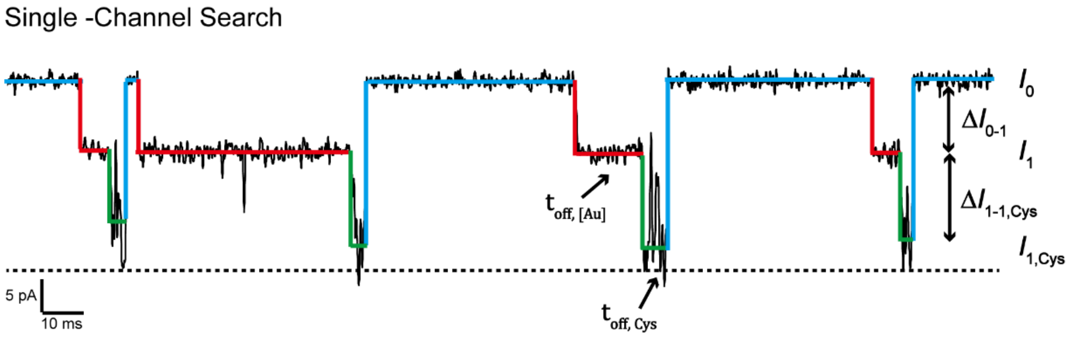

Supplementary Figure 16. Data analysis. All nanopore events ($[AuCl_4]^-$, Cys, Hcy or GSH) were extracted by the single-channel search feature of ClampFit, if not otherwise stated. The extraction result is demonstrated in this figure by taking a continuous trace of Cys sensing as an example **(Fig. 3d)**. The mean value of state 0, 1, 1_{SH} were marked with blue, red and green lines respectively. The definition of the event dwell time $t_{off,[Au]}$ and $t_{off,Cys}$ were marked on the figure. ΔI_{0-1} stands for the amplitude difference between I_0 and I_1. $\Delta I_{1-1,Cys}$ stands for the amplitude difference between I_0 and $I_{1,Cys}$.

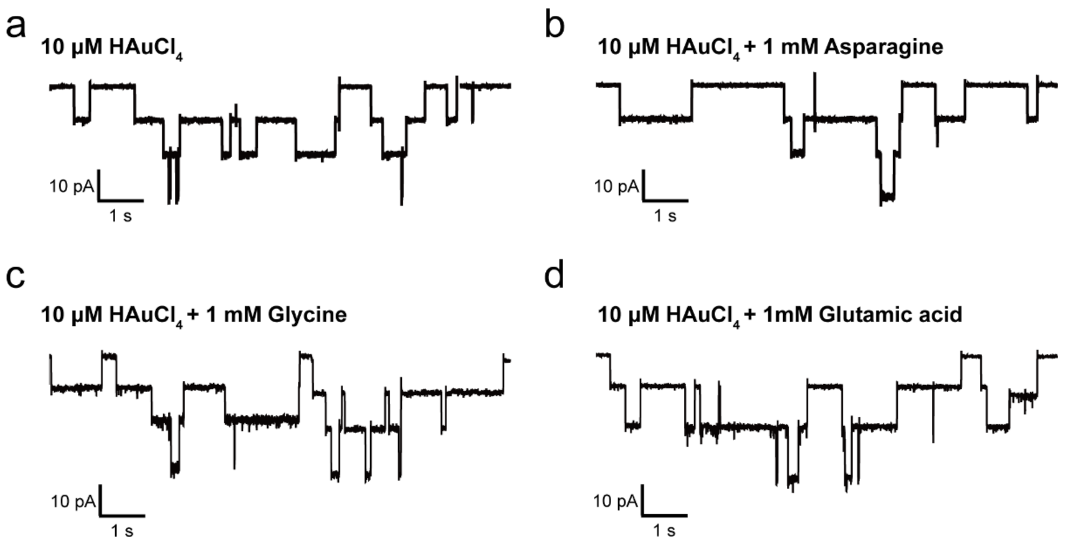

Supplementary Figure 17. MspA-M and amino acids other than biothiols. Electrophysiology recordings were performed with MspA-M when a +100 mV voltage was continuously applied and 10 μM $HAuCl_4$ was added in *cis*. **(a)** A representative current trace without the addition of any amino acids. Sequential binding of three $[AuCl_4]^-$ were clearly recognized, as described in **Supplementary Figure 7**. A representative current trace when recorded with a further addition of L-asparagine **(b)**, L-glycine **(c)**, L-glutamic acid **(d)** to *cis* reaching a 1 mM final concentration. As demonstrated in **b-d**, no clear sensing events, as observed from L-cysteine **(Fig. 3e)**, were observed for L-asparagine, L-glycine and L-glutamic acid. This indicates that a thiol group is critical in the generation of the event as demonstrated in **Fig. 3e**.

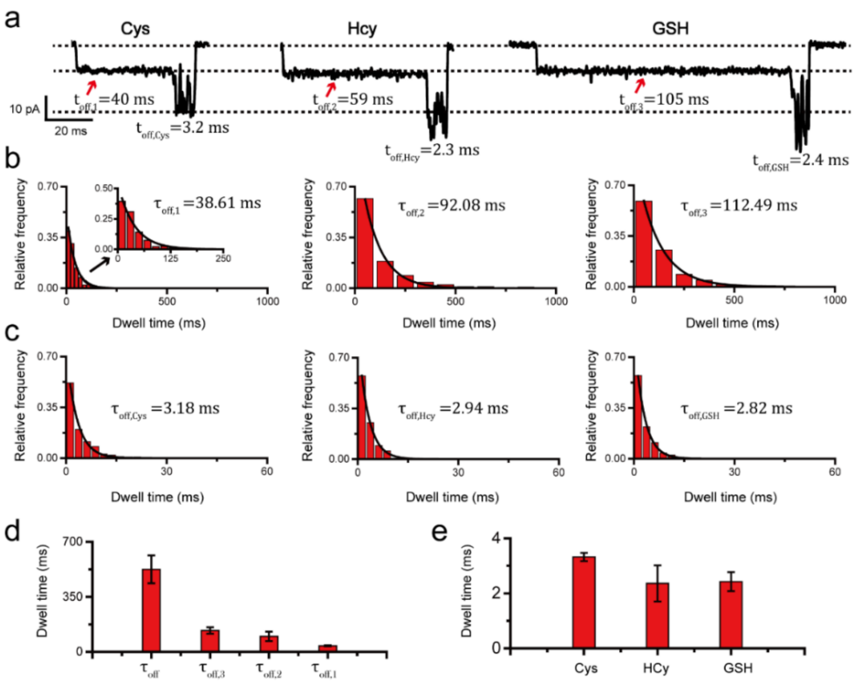

Supplementary Figure 18. Dwell time analysis. The statistical date were from electrophysiology recordings with MspA-M when 4 μM $HAuCl_4$ in *cis* and 40 μM Cys, Hcy or GSH in *trans* were present. **(a)** Representative blockage events of Cys, Hcy and GSH. The dwell time were marked on the event respectively. **(b)** Histograms of the $[AuCl_4]^-$ binding dwell time ($t_{off,1-3}$) when 40 μM Cys ($t_{off,1}$), Hcy ($t_{off,2}$) or GSH ($t_{off,3}$) were placed in *trans* respectively, as marked differently in the figure. Here $t_{off,1-3}$ stands for the dwell time of state 1 **(Fig. 3e)**. The mean dwell time ($\tau_{off,1-3}$)was derived from single exponential fitting results **(Supplementary Figure 8)**. **(c)** Histograms of the biothiols binding dwell time for Cys ($t_{off,Cys}$), Hcy ($t_{off,Hcy}$) or GSH ($t_{off,GSH}$). Here, $t_{off,Cys}$, $t_{off,Hcy}$ or $t_{off,GSH}$ stands for the dwell time of state 1_{SH} **(Fig. 3e)**. The mean dwell time τ_{off}were derived from single exponential fitting results **(Supplementary Figure 8)**. **(d)** Mean dwell time of $[AuCl_4]^-$ binding events. The $HAuCl_4$ concentration in *cis* were kept with 4 μM. τ_{off}, $\tau_{off,1}$, $\tau_{off,2}$ or $\tau_{off,3}$ stands for the dwell time of the $[AuCl_4]^-$ binding state when no biothiol, Cys, Hcy or GSH were added respectively. The binding of thiol-containing amino acids or peptides has significantly shortened the dwell time of $[AuCl_4]^-$ bound with the methionine around the pore restriction. This effect is most pronounced for Cys, followed by Hcy and GSH. All means and standard deviations were from three independent experiments for each condition (**Supplementary Table 5, 7, 9**). **(e)** The mean dwell time of Cys, Hcy and GSH bound in the pore. All means and standard deviations were from three independent experiments (10 min recording, N=3, **Supplementary Table 5, 7, 9**)

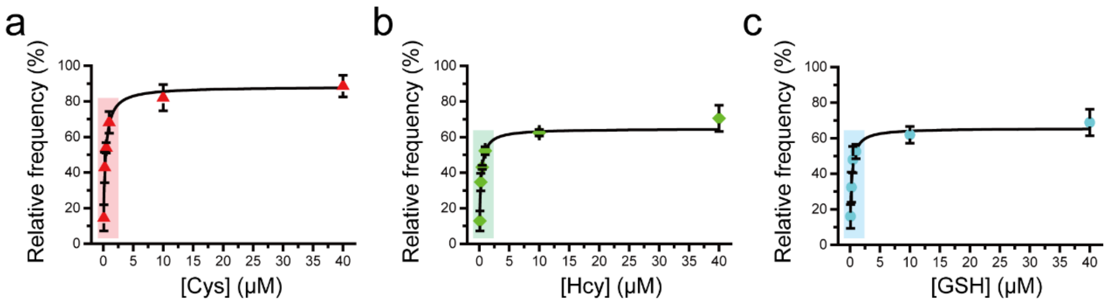

Supplementary Figure 19. Quantitative analysis of biothiols. As demonstrated in **Fig. 3, 4 and 5**, a proportion of $[AuCl_4]^-$ binding event contains signatures of biothiols binding. The relative frequency of biothiol events is thus defined as the count of biothiol event with respect to the count of all $[AuCl_4]^-$ containing event within a given period of recording. Experimentally, all nanopore measurements were performed as described in **Methods**. The concentration of chloroauric acid in cis was fixed at 4 μM and the concentration of biothiols (cysteine, homocysteine or glutathione) in trans was adjusted between 0-40 μM (0.1/0.25/500/1/10/40 μM). The relative frequency of **(a)** cysteine, **(b)** homocysteine and **(c)** glutathione binding was derived from all events acquired within a 10 min continuous recording for each condition and plotted versus the concentration of biothiols. Results in **(a-c)** indicates that the relative frequency of biothiols binding is dependent on the final concentration of biothiols, which could be fitted by the equation$\mathrm{y} = V_{max} * \mathrm{x}/(\mathrm{K}_m + \mathrm{x})$, indicating that the reaction follows the Michaelis-Menten model. According to the fitted curve, the relative binding frequency demonstrated in **(a-c)** shows a strong concentration dependence between 0-1 μM, which is marked in corresponding areas on the figure. Whereas, the relative binding frequency eventually saturates at a higher concentration of biothiols.

Supplementary Table 1. Statistics of ΔI_{0-1} measured with α-HL WT. The measurements were carried out as described in **Methods** with a +100 mV applied voltage and with 1 μM $HAuCl_4$ in *cis*. ΔI_{0-1} stands for the amplitude difference between I_0 and I_1. The mean dwell time (τ_{off}) was derived from single exponential fitting results as described in **Supplementary Figure 8**.

Independent experiments	Current blockade (ΔI_{0-1}) (pA)	Mean dwell time (τ_{off}) (s)
1	5.6 ± 0.3	11.15
2	4.7 ± 0.7	11.85
3	6.0 ± 1.0	9.58

Supplementary Table 2. Statistics of ΔI_{0-1} measured with MspA-M. The measurements were carried out as described in **Methods** with a +100 mV applied voltage and with 1 μM $HAuCl_4$ in *cis*. ΔI_{0-1} stands for the amplitude difference between I_0 and I_1. The mean dwell time (τ_{off}) was derived from single exponential fitting results as described in **Supplementary Figure 8**.

Independent experiments	Current blockade (ΔI_{0-1}) (pA)	Mean dwell time (τ_{off}) (ms)
1	11.3 ± 0.3	440.53
2	11.2 ± 0.5	427.35
3	12.5 ± 0.5	460.83

Supplementary Table 3. The association rate constant for $[AuCl_4]^-$ with MspA-M. Three independent measurements (N=3) were performed to form the statistics.

Concentration (μM)	$1/\tau_{on}$ (s^{-1})	Association rate constant ($M^{-1} s^{-1}$)
1	0.13 ± 0.02	
5	0.37 ± 0.25	$K_{on} = (8.61 \pm 4.11) \times 10^4$
10	0.52 ± 0.32	

Supplementary Table 4. Mean current blockade at different voltages. Single channel recordings were performed identically with either MspA-M or WT α-HL **(Methods)**. Briefly, $HAuCl_4$ was placed in *cis* with a 1 µM final concentration and varying voltages were applied during single channel recordings. By analyzing the current blockade (ΔI_{0-1}) amplitude for the tetrachloroaurate(III) binding events, MspA-M clearly outperform WT α-HL by systematically producing events with large amplitude.

Voltage (mV)	Current blockade (ΔI_{0-1}) for MspA-M (pA)	Current blockade (ΔI_{0-1}) for WT α-HL (pA)	Difference (pA)
20	0.90 ± 0.08	N.A.	N.A.
60	4 ± 1	2.2 ± 0.3	1.8
100	11.7 ± 0.7	5.4 ± 0.6	6.3
140	24.3 ± 0.7	11.6 ± 0.9	12.7
180	42.9 ± 0.5	18.3 ± 0.5	24.6
200	55 ± 2	24 ± 2	31

Supplementary Table 5. Statistics for L-Cysteine sensing. The statistical data were from 10 min continuous recordings with MspA-M when 4 μM $HAuCl_4$ were placed in *cis* and 40 μM Cys were placed in *trans*. A +100 mV voltage was applied. ΔI_{0-1} stands for the amplitude difference between I_0 and I_1. $\Delta I_{0-1,Cys}$ stands for the amplitude difference between I_0 and $I_{1,Cys}$. τ_{off} stands for the dwell time of I_1, $\tau_{off,Cys}$ stands for the dwell time of $I_{1,Cys}$. Three independent measurements were performed for each condition to form the statistics.

Independent experiments	Current blockade (ΔI_{0-1}) (pA)	Dwell time (τ_{off}) (ms)	Current blockade ($\Delta I_{0-1,Cys}$) (pA)	Dwell time ($\tau_{off,Cys}$) (ms)	$\Delta I_{1-1,Cys}$ (pA)
1	11.3 ± 0.5	35.51	25 ± 2	3.48	13.7
2	11.9 ± 0.7	38.61	25 ± 2	3.18	13.1
3	10.8 ± 0.3	41.23	25 ± 2	3.04	14.2

Supplementary Table 6. Statistics of $\Delta I_{0-1,Cys}/I_0$ for L-Cysteine sensing. The statistical data were from 10 min continuous recordings with MspA-M when 4 μM $HAuCl_4$ were placed in *cis* and 40 μM Cys were placed in *trans*. A +100 mV voltage was applied. ΔI_{0-1} stands for the amplitude difference between I_0 and I_1. $\Delta I_{0-1,Cys}$ stands for the amplitude difference between I_0 and $I_{1,Cys}$. Three independent measurements were performed for each condition to form the statistics.

Independent experiments	Open current (I_0) (pA)	$\Delta I_{0-1}/I_0$	$\Delta I_{0-1,cys}/I_0$
1	240	0.047 ± 0.002	0.104 ± 0.008
2	240	0.049 ± 0.003	0.105 ± 0.008
3	238	0.045 ± 0.002	0.104 ± 0.007

Supplementary Table 7. Statistics for L-Homocysteine sensing. The statistical data were from 10 min continuous recordings with MspA-M when 4 μM $HAuCl_4$ were placed in *cis* and 40 μM Hcy were placed in *trans*. A +100 mV voltage was applied. ΔI_{0-1} stands for the amplitude difference between I_0 and I_1. $\Delta I_{0-1,Hcy}$ stands for the amplitude difference between I_0 and $I_{1,Hcy}$. τ_{off} stands for the dwell time of I_1, $\tau_{off,Cys}$ stands for the dwell time of $I_{1,Hcy}$. Three independent measurements were performed for each condition to form the statistics.

Independent experiments	Current blockade (ΔI_{0-1}) (pA)	Dwell time (τ_{off}) (ms)	Current blockade ($\Delta I_{0-1,Hcy}$) (pA)	Dwell time ($\tau_{off,Hcy}$) (ms)	$\Delta I_{1-1,Hcy}$ (pA)
1	11.2 ± 0.3	92.08	33 ± 3	2.94	21.8
2	11.3 ± 0.5	130.72	34 ± 2	1.65	22.7
3	11.4 ± 0.5	71.94	35 ± 2	2.50	23.6

Supplementary Table 8. Statistics of $\Delta I_{0-1,Hcy}/I_0$L-Homocysteine sensing. The statistical data were from 10 min continuous recordings with MspA-M when 4 μM $HAuCl_4$ were placed in *cis* and 40 μM Hcy were placed in *trans*. A +100 mV voltage was applied. ΔI_{0-1} stands for the amplitude difference between I_0 and I_1. $\Delta I_{0-1,Hcy}$ stands for the amplitude difference between I_0 and $I_{1,Hcy}$. Three independent measurements were performed for each condition to form the statistics.

Independent experiments	Open current (I_0) (pA)	$\Delta I_{0-1}/I_0$	$\Delta I_{0-1,Hcy}/I_0$
1	265	0.042 ± 0.001	0.123 ± 0.01
2	265	0.043 ± 0.002	0.129 ± 0.01
3	266	0.043 ± 0.003	0.131 ± 0.01

Supplementary Table 9. Statistics for L-Glutathione sensing. The statistical data were from 10 min continuous recordings with MspA-M when 4 μM $HAuCl_4$ were placed in *cis* and 40 μM GSH were placed in *trans*. A +100 mV voltage was applied. ΔI_{0-1} stands for the amplitude difference between I_0 and I_1. $\Delta I_{0-1,GSH}$ stands for the amplitude difference between I_0 and $I_{1,GSH}$. τ_{off} stands for the dwell time of I_1, $\tau_{off,Cys}$ stands for the dwell time of $I_{1,GSH}$. Three independent measurements were performed for each condition to form the statistics.

Independent experiments	Current blockade (ΔI_{0-1}) (pA)	Dwell time (τ_{off}) (ms)	Current blockade ($\Delta I_{0-1,GSH}$) (pA)	Dwell time ($\tau_{off,Cys}$) (ms)	$\Delta I_{1-1,GSH}$ (pA)
1	10.8 ± 0.3	152.00	40 ± 6	2.19	29.2
2	10.9 ± 0.4	112.49	36 ± 3	2.82	25.1
3	10.7 ± 0.4	142.45	34 ± 4	2.27	23.3

Supplementary Table 10. Statistics of $\Delta I_{0-1,GSH}/I_0$ L-Glutathione sensing. The statistical data were from 10 min continuous recordings with MspA-M when 4 μM $HAuCl_4$ were placed in *cis* and 40 μM GSH were placed in *trans*. A +100 mV voltage was applied. ΔI_{0-1} stands for the amplitude difference between I_0 and I_1. $\Delta I_{0-1,GSH}$ stands for the amplitude difference between I_0 and $I_{1,GSH}$. Three independent measurements were performed for each condition to form the statistics.

Independent experiments	Open current (I_0) (pA)	$\Delta I_{0-1}/I_0$	$\Delta I_{0-1,GSH}/I_0$
1	238	0.045 ± 0.001	0.167 ± 0.023
2	235	0.046 ± 0.002	0.151 ± 0.015
3	235	0.049 ± 0.003	0.145 ± 0.015

Supplementary References

1. Wang Y*, et al.* Osmosis-Driven Motion-Type Modulation of Biological Nanopores for Parallel Optical Nucleic Acid Sensing. *ACS applied materials & interfaces* **10**, 7788-7797 (2018).

2. Choi LS, Mach T, Bayley H. Rates and stoichiometries of metal ion probes of cysteine residues within ion channels. *Biophysical journal* **105**, 356-364 (2013).
