# Supplementary material for: Giant single molecule chemistry events observed from a tetrachloroaurate(III) embedded Mycobacterium smegmatis porin A nanopore
Source: Nat Commun. 2019 Dec 11;10:5668. doi: 10.1038/s41467-019-13677-2 (PMC6906327; doi:10.1038/s41467-019-13677-2)
Supplement: Supplementary file 3 — Description of Additional Supplementary Files [file 41467_2019_13677_MOESM3_ESM.pdf]

Description of Additional Supplementary Files

Supplementary Movie 1. Reversible binding of [AuCl4]- with MspA-M. This video clip shows a continuous electrophysiology recording of tetrachloroaurate(III) binding with MspA-M. The electrophysiology recording was carried out as described (Method). HAuCl4 was added in cis with a 4 μM final concentration. With the addition of such a low concentration of HAuCl4, reversible binding of individual instead of multiple [AuCl4] - dominates (Fig. 1b). [AuCl4] - binding events show a highly consistent blockage amplitude, as demonstrated in the video. To show more event details, the video is played back with a 1/5 speed.

Supplementary Movie 2. Reversible and sequential binding of [AuCl4] - with MspA-M. This video clip shows a continuous electrophysiology recording of tetrachloroaurate(III) binding with MspA-M. The electrophysiology recording was carried out as described (Method). HAuCl4 was added in cis with a 10 μM final concentration. Reversible and sequential binding of multiple tetrachloroaurate(III) generates three event levels, which were named In (n=0-3) respectively as defined in Fig. 1c. Here n stands for the total number of tetrachloroaurate(III) simultaneously Nanjing University School of Chemistry and Chemical Engineering 163 Xianlin Ave, Nanjing, China 210023 Tel: 86 025-89681920/89681921 shuo.huang@nju.edu.cn Group website: http://hysz.nju.edu.cn/bionano bound in the pore (Fig. 1c). The events show a high consistency in the blockage amplitude. Only direct transition between states In and In±1 can be observed (Supplementary Figure 7). To show more event details, the video is played back with a 1/5 speed.

Supplementary Movie 3. L-Cysteine binding with Au(III) embedded MspA-M. This video clip shows a continuous electrophysiology recording of Cys binding with Au(III) embedded MspA-M. The electrophysiology recording was carried out as described (Method). 4 μM HAuCl4 was added in cis and 40 μM Cys was added in trans. An event of Cys binding is composed of three states (0, 1 and 1SH) as defined in Figure 3e. To show more event details, the video is played back with a 1/5 speed. The 2nd event in the demonstrated trace is a [AuCl4] - binding event (Fig. 1b), from which a jitter signal didn't appear. The rest of the events are Cys binding events (Fig. 3e). The event type is identified as described in Supplementary Figure 15.
